# Complete genome of a European hepatitis C virus subtype 1g isolate: phylogenetic and genetic analyses

**DOI:** 10.1186/1743-422X-5-72

**Published:** 2008-06-05

**Authors:** Maria A Bracho, Verónica Saludes, Elisa Martró, Ana Bargalló, Fernando González-Candelas, Vicent Ausina

**Affiliations:** 1 Institut "Cavanilles" de Biodiversitat i Biologia Evolutiva, Universitat de València, Paterna (València), Spain; 2 Servei de Microbiologia, Hospital Universitari Germans Trias i Pujol, Departament de Genètica i Microbiologia, Universitat Autònoma de Barcelona, Badalona, Barcelona, Spain; 3 Servei d'Aparell Digestiu, Hospital Universitari Germans Trias i Pujol, Departament de Genètica i Microbiologia, Universitat Autònoma de Barcelona, Badalona, Barcelona, Spain; 4 CIBER en Epidemiología y Salud Pública (CIBERESP), Spain; 5 CIBER en Enfermedades Respiratorias (CIBERES), Spain

## Abstract

**Background:**

Hepatitis C virus isolates have been classified into six main genotypes and a variable number of subtypes within each genotype, mainly based on phylogenetic analysis. Analyses of the genetic relationship among genotypes and subtypes are more reliable when complete genome sequences (or at least the full coding region) are used; however, so far 31 of 80 confirmed or proposed subtypes have at least one complete genome available. Of these, 20 correspond to confirmed subtypes of epidemic interest.

**Results:**

We present and analyse the first complete genome sequence of a HCV subtype 1g isolate. Phylogenetic and genetic distance analyses reveal that HCV-1g is the most divergent subtype among the HCV-1 confirmed subtypes. Potential genomic recombination events between genotypes or subtype 1 genomes were ruled out. We demonstrate phylogenetic congruence of previously deposited partial sequences of HCV-1g with respect to our sequence.

**Conclusion:**

In light of this, we propose changing the current status of its subtype-specific designation from provisional to confirmed.

## Background

Hepatitis C virus (HCV), a single-stranded positive-sense RNA virus belonging to the *Flaviviridae *family, is the leading etiologic agent of chronic liver disease. According to WHO, about 180 million people, an estimated 3% of the world population, are infected with HCV [1]. Its genome, which is approximately 9600 nucleotide (nt) long, contains two short untranslated regions at each end (5'UTR and 3'UTR) and a single ORF of about 9000 nt, known as polyprotein, encoding three structural (core, E1 and E2) and seven non-structural proteins (P7, NS2, NS3, NS4A, NS4B, NS5A and NS5B). Based mainly on phylogenetic analyses, all HCV isolates are currently grouped into six genotypes (from 1 to 6) [2], and within each genotype, closely related isolates cluster in a varying number of subtypes (designated with letters a, b, c and so on) [3]. Provisional designation of subtypes requires rigorous phylogenetic analysis of sequences from both the core/E1 region and the NS5B region obtained from three or more different infections. Confirmed designation status is acquired after intensive phylogenetic analysis including, at least, one complete genome sequence of the candidate subtype. Before a new subtype is confirmed, rigorous recombination and phylogenetic analyses should preclude both recombination events between subtypes and significant grouping within any of the confirmed subtypes [3].

Thirteen subtypes of HCV genotype 1 have been described so far (from 1a to 1m). However, only three subtypes (1a, 1b and 1c), for which the complete genome sequence has been obtained, have the status of confirmed subtype. The remaining subtypes (from 1d to 1m), from which only partial sequences are known, have been denoted as provisional. In addition, a complete genotype 1 sequence from an Equatorial Guinea isolate with unassigned subtype is also available [4].

The HCV genotype infecting a patient is important as it influences dose and duration of current antiviral therapy (pegylated alpha interferon plus ribavirin); patients infected with genotype 2 or 3 respond better than those infected with genotype 1 or 4 [5,6]. Apart from being an excellent method for reliable genotyping, phylogenetic and genetic analysis of appropriate sequence data, is an important tool for epidemiological surveys, including deep outbreak studies [7], novel transmission risks [8], viral evolution [9,10] and origin and spread of HCV epidemics [11-14].

In the present study, viral RNA was isolated from a specimen (serum) obtained from a 56-year-old Spanish female patient, who seroconverted to HCV after undergoing surgery and receiving a blood transfusion in 1996. No other recognizable risk factor could be identified for acquiring HCV infection. Serum was obtained before pegylated alpha interferon plus ribavirin treatment, to which the patient did not respond. Initially, HCV genotype was determined by means of two genotyping assays. First, an assay using the Trugene^® ^5'NC genotyping kit (TRUGENE 5'NC; Bayer HealthCare) based on the sequencing of a fragment of the 5'UTR, led to an ambiguous subtype 1a/1c. Secondly, use of the Abbott Real Time HCVTM kit (Abbott Diagnostics), which targets the NS5B region for genotype 1 but only distinguishes subtypes a and b, led to an unambiguous subtype 1a. Accurate identification as subtype 1g could only be determined after partial sequencing of the NS5B gene followed by both sequence comparison against sequence databases and phylogenetic analysis. Many authors have pointed out some discordant subtyping results on comparing the results obtained using different genotyping assays based on the 5'UTR [15-17] or on comparing results from these assays with results from genotyping in-house methods, based on NS5B sequences [18,19]. Furthermore, a more relevant point has definitively been demonstrated concerning the intrinsic limitations of the 5'UTR. Due to this region's high level of conservation, its power to reproduce phylogenetic trees obtained using complete genome is limited, and consequently, it fails to discriminate subtypes or even genotypes [20]. As a result of inefficient genotyping and subtyping in most commercial assays, the presence of some subtypes could have been underestimated, or some of them even ignored, in epidemiological investigations of circulating HCV variants. An important consequence of accurate assignation of HCV subtypes based on appropriate sequence data is that it turns routine genotyping into a reliable tool for molecular epidemiology studies in which, apart from a clear description of circulating subtypes, putative new subtypes and/or genotypes can be detected [21].

## Results and Discussion

Here we report the first complete genome of a hepatitis C virus subtype 1g isolate. To demonstrate this we have both performed phylogenetic analysis with representative complete genomes of all genotypes, including the confirmed subtypes 1a, 1b and 1c, and also with all of the partial subtype 1g sequences deposited in sequence databanks.

**Figure 1:**
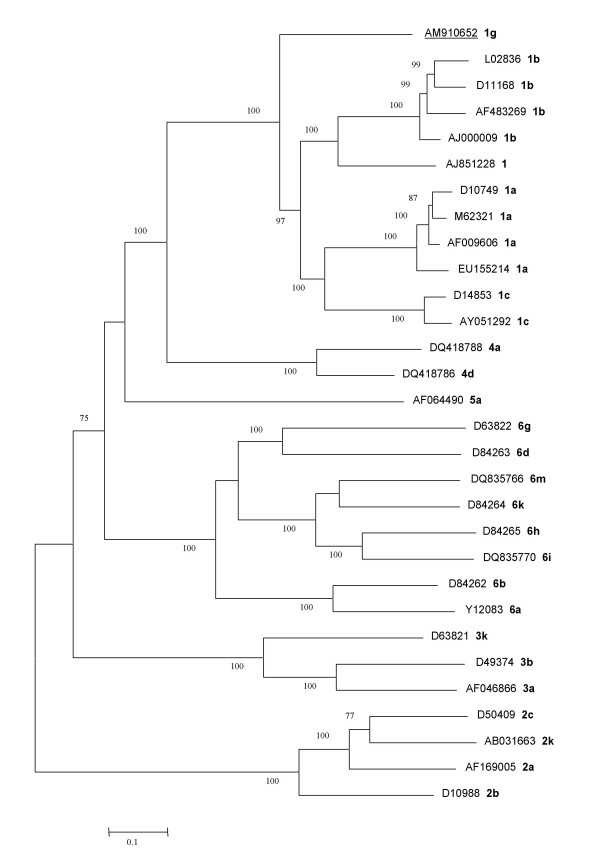
**Maximum likelihood phylogenetic tree of complete genome sequences**. Phylogenetic tree was obtained with PHYML using GTR+G+I for the new sequence and 29 complete genomes (coding region) representative of all 6 HCV genotypes. Genotype and subtype labels (in bold) are next to accession numbers. The sequence obtained in this study is underlined. Bar represents 0.1 substitutions per nucleotide position. Support value of nodes was estimated by bootstrap (1000 replicates using neighbour-joining with the maximum likelihood distance). Only values >75% are shown.

The complete subtype 1g genome (9490 nt) was obtained by direct sequencing of ten overlapping RT-PCR fragments, and includes the complete coding region and partial sequences from both 5'UTR and 3'UTR. A codon-based nucleotide alignment of the coding region of the new sequence was used in phylogenetic analyses, along with 29 representative sequences of all six HCV genotypes. In order to better represent genotype 1 subtypes, two or more sequences of subtypes 1a, 1b and 1c, were chosen. The best evolutionary model for this multiple alignment was determined according to the procedure implemented in Modeltest 3.8. This model, GTR+G+I, was used to obtain the unrooted maximum-likelihood phylogenetic tree shown in Fig. 1, in which the six well-defined clades corresponding to the six established genotypes were found, each containing all their known subtypes. It is worth noticing, in the tree showed, the significant grouping of genotypes 1 and 4 with a maximum bootstrap support. The close relationship between these two clades is only recognized in phylogenies using complete genomes where the nucleotide substitution model that best fits the data is taken into account [10,22]. Our subtype 1g sequence groups within the well-supported genotype 1 clade as a separate basal branch, which joins the group that includes all described HCV-1 subtypes. This indicates that divergence of subtype 1g occurred before evolutionary divergence of subtypes 1a, 1b and 1c.

Potential recombination events between genotypes or subtype 1 genomes were investigated following two approaches and, finally, ruled out. Firstly, phylogenetic reconstructions using the same representative sequences as in Fig. 1 were performed separately for the 10 protein-coding genes (from core to NS5B). With respect to the grouping of HCV-1g within HCV-1 subtypes, all the phylogenetic trees congruently reproduced the same topology obtained from the complete genome analysis. Moreover, subtype 1g still retained its basal position with respect to the other HCV-1 sequences in topologies based on E1, E2, NS2, NS4A, NS4B and NS5A genes (data not shown). Secondly, potential recombination events using the complete sequence alignment were investigated using the RDP 3.0b03 software [[23] and references therein]. This program implements several methods to identify of recombinant sequences and recombination breakpoints. All the recombination analyses based on the complete genome alignment showed no evidence that our subtype 1g sequence had participated in recombination events (data not shown).

The mean genetic distances between genotype 1 subtypes based on 26 representative sequences of subtypes 1a, 1b, 1c, an unassigned subtype 1 and our subtype 1g sequence were calculated (Table 1). The appropriate nucleotide substitution model was determined for this genotype 1 limited codon-based nucleotide alignment as described as above. The four mean genetic distances between the HCV-1g sequence and the other subtypes fall within the highest five of all comparisons. These results, based on complete genomes, suggest that the genotype 1g sequence is the most divergent genome with respect to the rest of the HCV-1 subtypes.

**Table 1:** Mean genetic distances among HCV subtype 1 representative sequences

	**subtype 1a**	**subtype 1b**	**subtype 1c**	**Unassigned subtype 1**	**subtype 1g**
**subtype 1a **(n = 10)		0.021	0.019	0.013	0.018
**subtype 1b **(n = 12)	0.690		0.019	0.014	0.012
**subtype 1c **(n = 2)	0.563	0.715		0.006	0.022
**Unassigned subtype 1 **(n = 1)	0.627	0.480	0.656		NA
**subtype 1g **(n = 1)	0.709	0.726	0.729	0.711	

NA, not applicable.

Mean genetic distances (lower-left matrix) among the HCV subtype 1g and representative sequences from subtypes 1a, 1b, 1c and an unassigned subtype 1 sequence. A codon-based nucleotide alignment containing the polyprotein of 26 complete genomes (9066 nucleotides) was used for distance estimates. Standard deviations are indicated in the upper-right matrix. Distance model was GTR+I+G with assumed proportion of invariable sites of 0.42 and a shape parameter (alpha) of 1.02 for the gamma distribution of substitution rates at variable sites. The number of sequences included in each subtype group is indicated in parenthesis.

**Figure 2:**
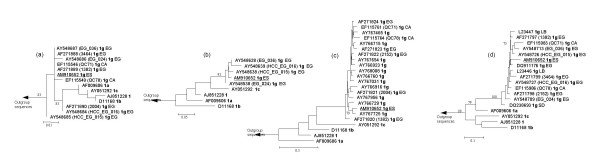
**Maximum likelihood phylogenetic trees including partial HCV-1g sequences**. Four regions were studied (a) 5'UTR, (b) core, (c) E1 and (d) NS5B. Only genotype 1 clade is shown. Support value of nodes was estimated by bootstrap (1000 replicates using neighbour joining with maximum likelihood distance). Only values >75% are shown. Bar represents in each case number of substitutions per nucleotide position. Patient code label (in parenthesis), genotype and subtype labels (in bold) are next to accession numbers. The sequence obtained in this study is underlined. Country names are CA, Canada; EG, Egypt; ES, Spain; LB, Lebanon and SD, Sudan.

Finally, we carried out phylogenetic analyses with our subtype 1g sequence and all available deposited sequences provisionally designated as subtype 1g (Fig. 2). These partial sequences, corresponding to four genomic regions (5'UTR, core, core/E1 and NS5B), were retrieved from the HCV sequence database in Los Alamos [24]. In all four analysed regions, the subtype 1g sequence described here always grouped within the sequences provisionally designated as subtype 1g. In eight cases, HCV genome from the same patient (the patient code appears in parenthesis in the corresponding trees) was partially sequenced in three different regions: 4 cases from Egypt (5'UTR, core and NS5B), 3 cases also from Egypt (5'UTR, core/E1 and NS5B) and 2 cases from Canada (5'UTR, core/E1 and NS5B). In all cases, we observed phylogenetic congruence of partial sequences of different regions obtained from the same specimen with respect to our subtype 1g sequence.

In the analyses of the NS5B region, three short deposited sequences were not included, because after nucleotide alignment the overlapping region was too short to be analysed. These three early deposited sequences, then considered subtype 1c and later assigned as subtype 1g, (Z70375, Z70392 and X88710) were obtained from sera dated between 1994 and 1995 in Germany [25] and would represent the first subtype 1g isolates detected in Europe. Although birthplace of these three patients could not be checked, the authors mentioned that some patients participating in the study had recently emigrated from Egypt and Sudan. The phylogenetic tree obtained using the NS5B region also includes 2 sequences from Lebanon [26], deposited in 1993 as subtype 1c and later assigned to subtype 1g (in fact, the two first subtype 1g sequences detected worldwide). The tree also includes one sequence from a Sudanese individual, detected in a study of unpaid blood donors in the Netherlands [27]. Interestingly, the patient in our study was born and resided in Spain, which is evidence of local transmission of subtype 1g.

## Conclusion

In summary, we have determined the complete genome sequence of an HCV-1g isolate, we have verified its grouping within HCV-1 and differentiation from other subtypes of this group by rigorous phylogenetic analyses, we have verified that this genome does not result from recombination events and that it is the most basal subtype among those belonging to HCV-1 for which a complete genome sequence is currently available. Taking this into account, we propose changing the status of subtype 1g from proposed to confirmed subtype.

## Methods

### Viral purification, RT-PCR and sequencing

Viral RNA was extracted from 200 μl of serum using High Pure Viral RNA Kit (Roche). Retrotranscription of viral RNA was performed in a final volume of 20 μl containing 10 μl of eluted RNA, 4 μl retrotranscription buffer, 500 μM of each dNTP, either 0.5 μg of random hexadeoxynucleotides (Promega) or 1 μM antigenomic sense primer, 100 U of M-MLV reverse transcriptase (Promega) and 20 U of rRNasin^® ^Ribonuclease Inhibitor (Promega). The mixture was incubated at 42°C for 60 min, followed by 3 min at 95°C. Table showed in additional file 1, lists the oligonucleotide primers used to obtain and/or sequence the overlapping RT-PCR products, which covered almost the whole genome. Primers denoted with "g" or "a" indicate genomic or antigenomic sense, respectively. Primers named with "h" refer to primers used in first round PCR followed by a hemi-nested PCR. Sequence primers named with "R" were directly designed from our sequence of subtype 1g. The genome was covered by 10 overlapping fragments using primer pairs: H28g-COA1a, COS2g-E1E2a, E1E2A2g-NS1a, NS1g2R-NS3a3, NS3g2R-305a2R, 1503g-577a, 5600gR-NS5a2R, KUg2-NS5B1a, NS5B1g-1279a, 1327gR-3utra2. When necessary, additional PCR fragments were also obtained by using combinations of the primers listed in additional file 1.

First round and hemi-nested amplifications were performed in a 50 μl volume containing either 5 μl of the RT product (in the case of first round PCR) or 1 μl of the first round PCR product (in the case of hemi-nested PCR), 5 μl of 10× PCR buffer, 100 μM of each dNTP, 200 nM of the genomic sense primer, 200 nM of the antigenomic sense primer and 5 U of Taq DNA Polymerase (Amersham). All PCRs were performed in a GeneAmp^® ^PCR system 2700 (Applied Biosystems) thermal cycler with the following profile: 95°C for 2 min, then 35 cycles at 95°C for 30 sec, 50–65°C (depending on the primers used) for 30 sec and 72°C for 3 min, and a final extension at 72°C for 10 min.

Amplified products were purified with High Pure PCR Products Purification Kit (Roche). These purified DNAs were sequenced using the ABI PRISM BigDye Terminator Cycle Sequencing Ready Reaction Kit v 3.1 in a 3700 automated sequencer (Applied Biosystems). Sequencing primers are also listed in additional file 1. Chromatogram files were assembled, verified and edited using the Staden Package [28]. The newly characterised sequence has been deposited in EMBL with accession number AM910652.

### Phylogenetic reconstructions and genetic distances

Two sets of nucleotide sequences were analysed: one corresponding to the complete polyprotein (Fig. 1) and the other corresponding to all sequences provisionally designed as subtype 1g and deposited at the HCV sequence database in Los Alamos [24] (Fig. 2). In the first set, the nucleotide sequence coding for the polyprotein (Fig. 1) was included in phylogenetic reconstructions along with 29 homologous complete genome sequences representative of the main HCV genotypes and subtypes (see accession numbers, genotypes and subtypes in Fig. 1). Selected sequences fulfil the condition of containing less than 15 ambiguities. In the second set, partial sequences belonging to four regions of HCV genome (5'UTR, core, core/E1 and NS5B) were analysed separately along with the corresponding homologous fragment of our subtype 1g complete genome. Alignments of partial sequences of HCV-1g used in phylogenetic reconstructions included (number of nucleotides in parenthesis): nine 5'UTR sequences (186 nt), four core sequences (217 nt), eighteen core/E1 sequences (220 nt) and thirteen NS5B sequences (222 nt). (see accession numbers, specimen name, and subtypes in Fig. 2). In addition, representative sequences used in the complete genome analysis for subtypes 2a, 3a, 4a, 5a and 6a (Fig. 1) and referred as outgroup were also included in the phylogenetic analyses.

ClustalW [29] implemented in MEGA version 4 [30] was used to obtain a multiple alignment of the corresponding amino acid sequences from which a codon-based nucleotide alignment was derived, except for the 5'UTR alignment. All phylogenetic trees were constructed by maximum likelihood in PHYML with the nucleotide substitution model that best fit the data according to Akaike Information Criterion (AIC) [31] for which we used the procedure implemented in Modeltest 3.8 [32]. The robustness of the tree topology was assessed by bootstrap analysis with 1000 replicates implemented in PHYML [33].

Estimates of mean distances between subtypes of HCV genotype 1 and between these and the new subtype 1g sequence were obtained with the maximum likelihood distance (see above) with PAUP*4.0b10 [34]. For this, we used 26 complete genomes from EMBL: our sequence for subtype 1g [EMBL: AM910652], ten sequences representing subtype 1a [EMBL: D10749, EMBL: M62321, EMBL: M67463, EMBL: AF009606, EMBL: AF011751, EMBL: AF011752, EMBL: AF290978, EMBL: AF271632, EMBL: AJ278830, EMBL: EU155214], twelve sequences for subtype 1b [EMBL: D11168, EMBL: D14484, EMBL: D45172, EMBL: L02836, EMBL: AB080299, EMBL: AB016785, EMBL: AB049095, EMBL: AF139594, EMBL: AF165048, EMBL: AF333324, EMBL: AJ000009, EMBL: AY045702), two sequences for subtype 1c [EMBL: D14853, EMBL: AY051292] and one sequence that corresponds to an unassigned subtype 1 [EMBL: AJ851228].

## Competing interests

The authors declare that they have no competing interests.

## Authors' contributions

MAB, VS, and EM co-conceived, designed and coordinated the study, participated in the molecular studies, sequence alignment, phylogenetic and genetic analyses, interpreted data, and co-drafted the manuscript; AB and VA performed the clinical work, recruitment of the patient, procurement of specimens and participated in proofreading of the manuscript; FG-C coordinated the study, interpreted data, co-performed phylogenetic and genetic analyses and participated in proofreading of the manuscript. All authors read and approved the final manuscript

## Supplementary Material

Additional file 1 Oligonucleotide primers used for amplification and sequencing. List of oligonucleotide primers including name, sequence, position and sense. Click here for file
