# Supplementary material for: Complete genome of a European hepatitis C virus subtype 1g isolate: phylogenetic and genetic analyses
Source: Virol J. 2008 Jun 5;5:72. doi: 10.1186/1743-422X-5-72 (PMC2438343; doi:10.1186/1743-422X-5-72)
Supplement: Additional file 1 — Oligonucleotide primers used for amplification and sequencing. List of oligonucleotide primers including name, sequence, position and sense. [file 1743-422X-5-72-S1.doc]

Sequence of oligonucleotide primers used for amplification and sequencing of overlapping regions of the subtype 1g sequence. Nucleotide position is relative to reference sequence H77 (accession number AF009606). Primers in bold were used for both PCR amplification and sequencing.

| Primer | Sequence 5’-3’ | Position | Sense |
| --- | --- | --- | --- |
| **H28g** | CCGATTGGGGGCGACACTCC | 9 to 28 | genomic |
| 348g | GCGACACTCCRCCAT | 19 to 33 | genomic |
| H3a | CGAGACCTCCCGGGGCACTCGCAAGCACCC | 299 to 328 | antigenomic |
| H5a | CTATCAGGCAGTACCACAAGGCCTTTCGCG | 270 to 299 | antigenomic |
| **COA1gh** | GGGAGGTCTCGTAGACCGTGCAYCATG | 318 to 341 | genomic |
| **COS2g** | AGACCGTGCAYCATGAGCAC | 330 to 349 | genomic |
| 264g | GCGGGATGGCTCYTGTC | 621 to 637 | genomic |
| COS2a | TACGCCGGGGGTCBRTRGGRCCCCA | 660 to 684 | antigenomic |
| 348a | TGACCTTACCCAAATTRCG | 690 to 708 | antigenomic |
| **COA1a** | RASSGGRATGTACCCCATGAGGTCGGC | 732 to 758 | antigenomic |
| CO1g | TACATTCCGCTCGTCG | 747 to 762 | genomic |
| COR2a | CRGGVARRTTCCCTGTTG | 835 to 852 | antigenomic |
| **E1E2A1gh** | CGYATGGCYTGGGAYATGAT | 1290 to 1309 | genomic |
| **E1E2A2g** | GGGAYATGATGATGAAYTGGTC | 1300 to 1321 | genomic |
| E1a | CCACCATGTCCACGAT | 1371 to 1386 | antigenomic |
| H6a | CCCGCYARGAYNCCCCARTGG | 1394 to 1414 | antigenomic |
| H7a | GCCCAGTTYCCYRYCATVGA | 1428 to 1447 | antigenomic |
| H11g | TCBATGRYRGGRAACTGGGC | 1428 to 1447 | genomic |
| **E1E2a** | GGGGTGAARCARTAYACTGG | 1854 to 1873 | antigenomic |
| **NS1g1hR** | CCGGCCTGATCCACCTGCAC | 2401 to 2420 | genomic |
| **NS1g2R** | GATCCACCTGCACCAAAACAT | 2408 to 2428 | genomic |
| **NS1a** | TASARGTAYTGYAYRTCCAC | 2430 to 2449 | antigenomic |
| H14gR | ATGGCACTGCCCCACCGGGC | 2742 to 2761 | genomic |
| H14g | ATGGACCSRGARRTGGCYGCR | 2769 to 2789 | genomic |
| H12a | CGCCYCCRCAYGAYGCRGCCAY | 2781 to 2802 | antigenomic |
| NS2a2 | nadyttggtgatktcaaa | 2997 to 3014 | antigenomic |
| **NS3g1h** | gcVgMBtgYggDgaYatcat | 3312 to 3331 | genomic |
| NS3a1R | ATGATGTCGCCACACGCCGC | 3312 to 3331 | antigenomic |
| **NS3g2R** | GACCAAGACCTCGTAGGGTG | 3654 to 3673 | genomic |
| NS3a1 | GAGRHGCCGYANGTGCA | 3708 to 3724 | antigenomic |
| 305g | YTRGTCACSAGRCAYGC | 3735 to 3751 | genomic |
| NS3a4 | gccggsacyttvgtgct | 4050 to 4066 | antigenomic |
| **NS3a3** | GCYGCRTANGCRGCCGG | 4062 to 4078 | antigenomic |
| **1503g** | GGVAGRCATCTYATYTTCTG | 4503 to 4522 | genomic |
| **305a2R** | TGCGCTTCGAGTGACAGA | 4519 to 4536 | antigenomic |
| 305-1gR | ACTCGAAGCGCAAGTGCG | 4525 to 4542 | genomic |
| 4700gR | GACTCAGTGATAGACTGCAA | 4686 to 4705 | genomic |
| Ns3-6a | AAGGTAGGGTCAAGGCTGAA | 4731 to 4750 | antigenomic |
| 577g | CYTGGTAYGAGCTBACRC | 4918 to 4935 | genomic |
| H17a | GACGACCTCYARRTCRGCYGM | 5289 to 5309 | antigenomic |
| **5600gR** | GCTCCCATCGTGCAATCCAA | 5141 to 5160 | genomic |
| 472g | TGGCYGCDTAYTGYCT | 5350 to 5365 | genomic |
| 577gR | GGAACTTCATCAGCGGCAT | 5638 to 5656 | genomic |
| **577a** | atsccrctraygaarttcc | 5656 to 5638 | antigenomic |
| **472ah** | gcwatyaghcggttcatc | 6186 to 6103 | antigenomic |
| **KUg1h** | tggayggrgtrcggytgcacaggta | 6715 to 6739 | genomic |
| **KUg2** | CAGGTACGCTCCRGYRTGCA | 6734 to 6753 | genomic |
| KUaR | GAGGCTTGCATGGCGGGGCG | 6740 to 6759 | antigenomic |
| **NS5a1R** | gaccacataggagttgag | 6780 to 6797 | antigenomic |
| **NS5a2hR** | ataggagttgaggccaac | 6786 to 6803 | antigenomic |
| 780a2 | TCRAGGGGRGGCATRGAGGA | 7497 to 7516 | antigenomic |
| 780a1 | CCYTCRAGGGGGGGCAT | 7503 to 7519 | antigenomic |
| H22g | CAGYGAYGGGTCYTGGTCYAC | 7541 to 7561 | genomic |
| 830g | GYNTGCTGYTCRATGTC | 7593 to 7609 | genomic |
| **NS5B1g** | TATGATACYCGCTGYTTYGACTC | 8256 to 8278 | genomic |
| 830a | gagtcaaarcarcgggtr | 8261 to 8278 | antigenomic |
| NS5g2 | AAAGCTCCAGGACTGCA | 8519 to 8535 | genomic |
| **NS5B1a** | GTACCTRGTCATAGCCTCCGTGAA | 8616 to 8639 | antigenomic |
| **1327gR** | GGAAACAGCTAGACACACTC | 8792 to 8811 | genomic |
| 1327g | acagyhmgrcacactc | 8796 to 8811 | genomic |
| **1279a** | tKatRttKccYaRccagga | 8838 to 8820 | antigenomic |
| H24g | TCTACGGRGCYDNYTACTCCATT | 8941 to 8963 | genomic |
| **H27ah** | GTCAAGTGGYTCAATGGAGTARNHRGC | 8949 to 8975 | antigenomic |
| Utr3g1 | CGGCTACAGCGGGGGAGACAT | 9260 to 9280 | genomic |
| Utr3g2 | ACAGCGGGGGAGACATWTATC | 9265 to 9285 | genomic |
| Ns5a4 | tgatawatgtctccccc | 9270 to 9286 | antigenomic |
| Ns5Butr3g | GGGGGAGACATWTATCACAGC | 9270 to 9290 | genomic |
| Ns5a3 | CASGCTGTGATAWATGTC | 9276 to 9293 | antigenomic |
| 3utra | gmragyaggagtaggca | 9324 to 9340 | antigenomic |
| 1327a | AGSARRWARATGCCTACC | 9347 to 9364 | antigenomic |
| **3UTRa2** | agcactctctgcagtcatgcgg | 9599 to 9620 | antigenomic |
| **H26a1h** | CRGCAMTCYCTGCRGTCA | 9621 to 9604 | antigenomic |
